# Multiday EMG-Based Classification of Hand Motions with Deep Learning Techniques

**DOI:** 10.3390/s18082497

**Published:** 2018-08-01

**Authors:** Muhammad Zia ur Rehman, Asim Waris, Syed Omer Gilani, Mads Jochumsen, Imran Khan Niazi, Mohsin Jamil, Dario Farina, Ernest Nlandu Kamavuako

**Affiliations:** 1 Department of Robotics & Artificial Intelligence, School of Mechanical & Manufacturing Engineering, National University of Sciences & Technology (NUST), Islamabad 44000, Pakistan; aw@hst.aau.dk (A.W.); omer@smme.nust.edu.pk (S.O.G.); mohsin@smme.nust.edu.pk (M.J.); 2 Center for Sensory-Motor Interaction, Department of Health Science and Technology, Aalborg University, 9200 Aalborg, Denmark; mj@hst.aau.dk (M.J.); imran.niazi@nzchiro.co.nz (I.K.N.); 3 Center for Chiropractic Research, New Zealand College of Chiropractic, Auckland 1060, New Zealand; 4 Health and Rehabilitation Research Institute, AUT University, Auckland 1142, New Zealand; 5 Department of Electrical Engineering, Faculty of Engineering, Islamic University Medina, Medina 41411, Saudi Arabia; 6 Department Bioengineering, Imperial College London, London SW72AZ, UK; d.farina@imperial.ac.uk; 7 Centre for Robotics Research, Department of Informatics, King’s College, London WC2G4BG, UK; ernest.kamavuako@kcl.ac.uk

**Keywords:** myocontrol, pattern recognition, electromyography, autoencoders, convolutional neural networks, multiday classification, MYO band

## Abstract

Pattern recognition of electromyography (EMG) signals can potentially improve the performance of myoelectric control for upper limb prostheses with respect to current clinical approaches based on direct control. However, the choice of features for classification is challenging and impacts long-term performance. Here, we propose the use of EMG raw signals as direct inputs to deep networks with intrinsic feature extraction capabilities recorded over multiple days. Seven able-bodied subjects performed six active motions (plus rest), and EMG signals were recorded for 15 consecutive days with two sessions per day using the MYO armband (MYB, a wearable EMG sensor). The classification was performed by a convolutional neural network (CNN) with raw bipolar EMG samples as the inputs, and the performance was compared with linear discriminant analysis (LDA) and stacked sparse autoencoders with features (SSAE-f) and raw samples (SSAE-r) as inputs. CNN outperformed (lower classification error) both LDA and SSAE-r in the within-session, between sessions on same day, between the pair of days, and leave-out one-day evaluation (*p* < 0.001) analyses. However, no significant difference was found between CNN and SSAE-f. These results demonstrated that CNN significantly improved performance and increased robustness over time compared with standard LDA with associated handcrafted features. This data-driven features extraction approach may overcome the problem of the feature calibration and selection in myoelectric control.

## 1. Introduction

Myoelectric control for upper limb prostheses is based on the electrical activity (electromyogram, EMG) generated in remnant muscles, a technology that dates back to 1948 [1]. This approach is commonly used in clinical prosthetic systems [2]. Commercially available upper limb electric prostheses use conventional myoelectric control schemes, such as on/off, proportional, and direct activation [3] and are often limited to one degree of freedom (DoF) [4]. Pattern recognition (PR)-based control schemes have emerged as an alternative to conventional myoelectric control schemes for activation of multiple DoFs [5]. These systems have been widely explored to improve the multifunctionality of dexterous prosthetic hands [6,7,8,9], yet their clinical usability is still limited.

The two important steps in PR-based schemes are feature extraction and classification. The choice of optimal features for classification is a challenging task [10,11,12,13,14]. Hudgins et al. [11] proposed four time-domain features that have been subsequently extensively used and are now considered a benchmark. EMG signals are stochastic in nature, and their statistical properties may change over time, even within the same recording session. For example, changes in arm posture and fatigue influence the EMG, among several other factors [6]. These factors influence the features and decrease the performance of the systems over time [15]. Phinyomark [12,16,17] compared several alternative time and frequency-domain features individually and in combination. However, these features have so far not shown substantial advantages over the simpler time-domain features and, specifically, not in terms of the robustness of the system. Furthermore, the optimization of a single or a combination of the handcrafted EMG features [18] has proven not to be efficient in providing the adequate and robust controllability. Therefore, the data-driven automatic feature selection has been proposed to improve robustness [19,20,21].

Following feature selection, classification has been performed with several approaches, including linear discriminant analysis (LDA) [22], support vector machine (SVM) [23], artificial neural networks (ANN) [24], hidden Markov model [25], decision tree [26], Bayesian network [27], k-nearest neighbor (KNN), and random forests (RF) [28]. However, the selection of features is challenging. In the last decade, deep learning algorithms have shown promising results in several fields, including computer vision [29], natural language processing [30], speech recognition [31], and bioinformatics [32]. These algorithms are the combinations of many non-linear layers of ANN with the capability of driving data-dependent features from the raw data. Convolutional neural networks have been applied for myoelectric control with the focus on intersessions/subjects and intrasession performance, in addition to many other applications in biomedical signal processing [33,34,35]. For intersessions/subjects, some authors have used the adaptation strategy from previous sessions, while others have used separate sessions as training and testing data. These works are being performed on publicly available databases, including Ninapro [21,36], Capgmyo, and csl-hdemg [37].

### 1.1. Deep Learning for Myoelectric Control

In the pioneer work of CNN-based myoelectric control schemes, Park and Lee [38] developed a user-adaptive multilayer CNN algorithm to classify surface (sEMG) patterns using data from the Ninapro database and found that CNN outperformed SVM in both the non-adaptation and adaptation scheme by about 12–18 percentage points. Geng et al. [37] proposed a deep convolutional network for high-density sEMG images. They evaluated their proposed algorithm on all three databases (mentioned above) and found that deep networks outperformed the classical classifiers including KNN, SVM, LDA, and RF. However, Atzori et al. [21] showed that CNN performance was comparable to other classical classifiers, including KNN, SVM, and LDA when using a modified version of the well-known CNN architecture called LeNet [39]. Du et al. [40] presented a benchmark high-density sEMG (HDsEMG) database and developed a multilayer CNN based on a deep domain adaptation framework. They performed both the intra- and intersession/subject analysis and found that deep domain adaptation-based architecture outperformed all the other classical classifiers. Zhai et al. [15] claimed to propose a CNN-based self-recalibrating classifier that could update over time but the dataset was within the same day. They found that it significantly outperformed the SVM classifier. Du et al. [41] proposed a semi-supervised learning algorithm based on CNN for unlabeled data and used data glove to learn additional information about hand postures and temporal orders of sEMG frames. They found that classification accuracy improved significantly as compared with random forests, AtzoriNet [21], and GengNet [37]. Wei et al. [42] proposed a multi-stream CNN with decomposition and fusion stages that could learn the correlation between individual muscles. They used the divide and conquer strategy and evaluated their model on three benchmark databases. The results showed that multi-stream CNN outperformed the simple CNN and random forests classifiers. Xia et al. [20] proposed for the first time a hybrid CNN–RNN (recurrent neural network) architecture to address the variation in signals over time. The input to this network consisted of time-frequency frames from sEMG signals and evaluated it with the data of eight subjects recorded in six sessions, though on the same day. The results showed that the hybrid CNN–RNN architecture outperformed CNN and support vector regression (SVR). Allard et al. [19] performed a real-time study with transfer learning based on CNN. They collected two different datasets of 18 and 17 subjects, respectively, using an eight-channel MYO armband (a wearable EMG sensor) and controlled a six-DoF robotic arm. Their proposed CNN achieved 97.8% accuracy for seven hand movements, slightly better than the baseline CNN (96.2%). Using classification accuracy as a performance metric, these studies have shown that deep learning techniques are promising for myoelectrical control schemes. However, they were performed on datasets that were collected in single or multiple sessions (short-term) within the same day. Although the idea of a self-adaptive algorithm is noble and encouraging, robustness over time, with day-to-day variations and potential adaptation from the user, have remained unexplored.

### 1.2. Contribution

The use of machine learning (ML), rather than direct control, has attempted to advance the control possibilities of the users [6], but it is limited by unsatisfactory robustness to non-stationarities (e.g., changes in electrode positions and the skin–electrode interface) [43]. Robustness is the key characteristics of any clinical solution. Very advanced control systems, including recently proposed deep networks that allow a substantial functional benefit for short-term laboratory tests, cannot be translated into clinical solutions if their performance worsens over time. The long-term performance of conventional ML algorithms relies heavily on data representations called features. Thus, the problem of robustness is associated with reliable performance over time and the choice of features to describe the signal for subsequent classification. The above literature review revealed that the proposed deep learning algorithms make use of short-term recordings with prior data transformation, reducing the EMG signal into a handcrafted feature and making the problem similar to a conventional ML approach. Furthermore, in such short-term conditions, the need for deep learning with respect to more conventional ML methods is very limited, because conventional ML has been shown to be very effective (classification accuracy easily >95% for >10 classes [44]) in short laboratory recordings. Recently, it has been shown that classification accuracies vary significantly over time [45,46,47], as data recorded on one day has different characteristics from data recorded on another day due to real-world conditions. The key challenge is not the laboratory, short-term conditions but daily use. Thus, we propose a longitudinal approach to myoelectric control that makes use of a convolutional neural network architecture with raw EMG signals as inputs, in order to explore the real potential of deep learning in utilizing the intrinsic features (deep features) of the EMG signals, specifically to enhance the long-term robustness of the classification task.

Adaptive learning strategies for classifiers are promising [15,48], but this work focused on the effect of long-term bipolar EMG data recording on performance and did not explore algorithm adaptation to avoid confusion over the changes observed in performance, if there were any, were due to longitudinal data and not because of the adaptive algorithm. The EMG signals were recorded in two experimental sessions per day over 15 consecutive days. We have evaluated the performance of stacked sparse autoencoders (SSAE), an unsupervised deep learning technique, with both handcrafted features (SSAE-f) and raw EMG samples (SSAE-r) extracted from varying lengths (1–15 days) or recorded EMG. Intrasession, intersession, and inter-days analyses were performed, and the performance of both the CNN and SSAE were compared with state-of-the-art LDA.

## 2. Materials and Methods

### 2.1. Subjects

Seven able-bodied subjects (four males and three females, age range of 24–30 years, and mean age 27.5 years) participated in the experiments. They had no known prior history of musculoskeletal or upper extremity disorders, and their right hand was used in the experiments. The procedures were in accordance with the Declaration of Helsinki and approved by the local ethical committee of Northern Jutland (approval no: N–20160021). All the subjects participated voluntarily and provided written informed consent prior to the experimental procedures.

### 2.2. Wearable EMG Sensors

The data were recorded using the commercial MYO Armband (MYB). MYB are wearable EMG sensors that are developed by Thalamic Lab (Kitchener, ON, Canada, https://www.myo.com/) and have eight channels of dry electrodes with a sampling frequency of 200 Hz. It is a low-cost, consumer-grade device with a nine-axis inertial measurement unit (IMU) [26] that can communicate wirelessly with PCs via Bluetooth. It is a non-invasive, more user-friendly and time-saving device compared with conventional electrodes [49,50]. Notwithstanding the low sampling frequency, its performance has been shown to be similar to full-band EMG recordings using conventional electrodes [51,52], and the technology has been used in many studies [53,54,55,56,57,58,59,60,61,62,63,64]. Therefore, this study did not focus on the comparison with conventional EMG electrodes.

### 2.3. Experimental Procedure

**Figure 1:**
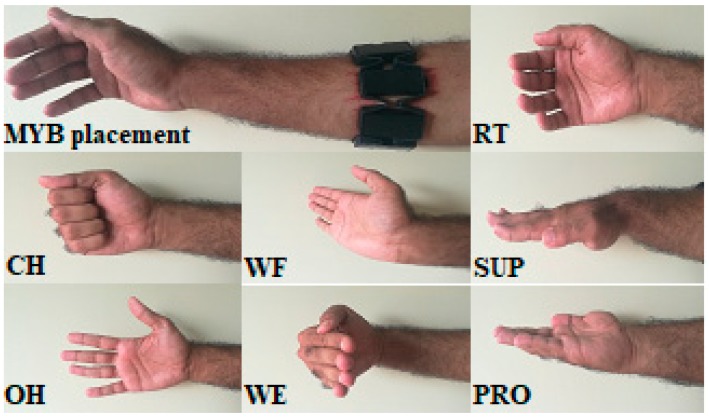
Position of the MYO armband (MYB) on the forearm and different types of motions considered in this work. RT: rest; CH: close hand; WF: wrist flexion; SUP: supination; OH: open hand; WE: wrist extension; and PRO: pronation.

The MYB was worn over the forearm and placed approximately three centimeters distal to the elbow crease and the olecranon process of the ulna, where it covered the surface of the extensor carpi radialis, extensor digitorum, extensor carpi ulnaris, flexor carpi radialis, palmaris longus, and flexor digitorum superficialis muscles, as shown in Figure 1.

The protocol was designed such that each subject performed seven movements (each movement was shown to the subject using a custom-made graphical user interface (GUI) and lasted for 6 s) with 10 repetitions per movement in a single session. The data were recorded for 15 consecutive days, and two sessions were recorded per day with a break of one hour in between. Markers were placed to ensure the correct MYB placement for each session for consecutive days. The hand movements included close hand (CH), open hand (OH), wrist flexion (WF), wrist extension (WE), pronation (PRO), supination (SUP), and rest (RT), as shown in Figure 1. Each movement was repeated with a contraction and a relaxation period of 4 s each. The sequence of the movements was randomized for each session.

### 2.4. Signal Processing

The data was filtered with a third-order Butterworth high-pass filter with a cut-off frequency of 2 Hz to reduce movement artifacts. Overlapping windows of 150 ms with a step of 25 ms were extracted. For CNN- and SSAE-r-based classification, the raw EMG samples were directly used as inputs (size = 30 × 8), while for SSAE-f- and LDA-based classification, four time-domain features (TDFs) were extracted (size = 4 × 8): mean absolute value (MAV), waveform length (WL), slope sign change (SSC), and zero crossing (ZC). The choice of these TDFs and this particular machine learning algorithm (LDA) was made as most MYB-based studies [51,60] applied this combination, and recent studies [59,65] have shown that LDA achieved the highest accuracies with these TDFs as compared with ANN, SVM, KNN, RF, and naïve Bayes (NB). Furthermore, LDA is now also being used with a commercial prosthetic hand COAPT [66] (Coapt, Chicago, IL, USA, https://www.coaptengineering.com). Zero-threshold value was used for SSC and ZC features [67].

The details of SSAE and CNN are discussed in Section 2.5 and Section 2.6, while LDA was used from a publicly available EMG processing library (MECLAB) [22]. In order to quantify the short-term and long-term performance of the classifiers, both within-day and between-days analyses were performed for each subject. The results are presented as means across all subjects. 

The within-day analysis included within session with 10-fold validation (because there are 10 repetitions per session) and between sessions with two-fold validation (because there are two sessions per day), while between-days analysis included two-fold validation (between pairs of days and hence two-fold validation was used) and k-fold cross-validations (k = 15 days and hence 15-fold validation was used) in a leave-one-out fashion. The classification error (CE), defined as the number of samples wrongly classified divided by the total number of samples, was used as a performance metric. This is related to the classification accuracy (CA) as CA = 1 − CE. Both metrics are widely used to quantify performance in offline myoelectric control studies. 

### 2.5. Autoencoders

Autoencoders are unsupervised deep networks [68] in which input signals are encoded to a new representation and constructed back at the output via decoders. The error between the original input and the reconstructed input is minimized using criteria such as L2 regularization (L2R), sparsity proportion (SP), and sparsity regularization (SR), and hence, it optimizes the new representation of the data (data-driven features).

**Figure 2:**
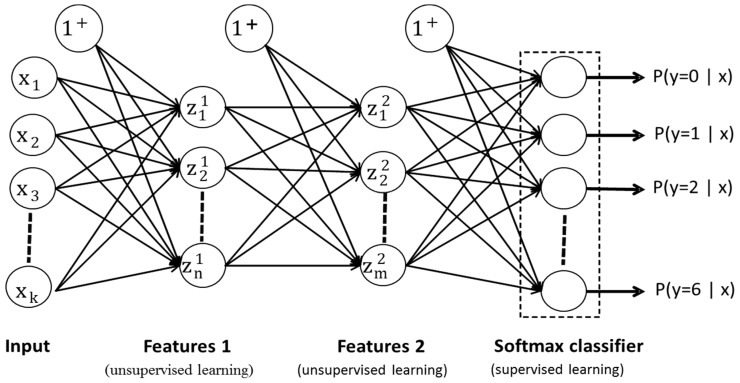
Block diagram of stacked sparse autoencoders (SSAE). The features are learned in an unsupervised way, while classification is performed in the supervised fashion. The lengths of both layers were adjusted accordingly to input the lengths of SSAE-f (k = 4 × 8, n = 32, m = 6) and SSAE-r (k = 30 × 8, n = 100, m = 50).

In this work, two-layer SSAE were used as previously described [46,69,70] and as shown in Figure 2. The network was trained with the scale conjugate gradient descent algorithm [71] using greedy layer-wise training [72]. The parameters were optimized as previously detailed [46], and the length of layers was adjusted for both SSAE-f and SSAE-r. For SSAE-f, the sizes of the first and the second layers were 32 (k = n) and 16 units, respectively, while for SSAE-r, they were 100 (k > n) and 50 units, respectively, such that any further increment in the sizes of layers (n, m) just increased the computational cost. The final parameters’ values for both layers were set as follows. L2 regularization (L2R) was set to 0.0001, sparsity regularization (SR) to 0.01, and sparsity proportion (SP) to 0.5.

### 2.6. Convolutional Neural Networks

The CNN is an important architecture of deep learning that is a modification of ordinary neural networks for processing multiple arrays of data, such as images, signals, and language. They work on three simple ideas, including local receptive field, shared weights, and pooling [68]. In the convolutional layer, filters are convolved with patches of input (receptive field) such that an individual filter shares the same learning weights for all patches. The dot product of filters with patches is passed through the activation unit, and the size of output is reduced via pooling.

**Figure 3:**
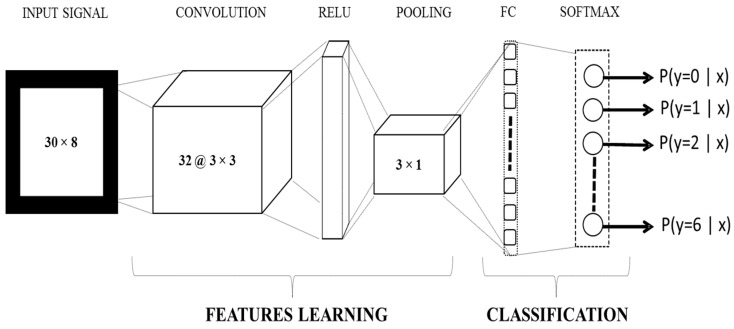
Block diagram of the convolutional neural network (CNN) used in this work. The input corresponds to a 150 ms (30 × 8 samples) window of eight channels. There were only single layers of convolution, Relu, pooling, and fully connected (FC) layers, while Softmax was used for classification.

In this work, single-layer CNN architecture (as shown in Figure 3) is implemented using the neural network toolbox in MATLAB 2017a. The input corresponds to 150 ms (30 × 8 samples) bipolar raw EMG data of eight channels. The convolutional layer includes 32 filters of size 3 × 3, a Relu layer, a max pooling layer of size 3 × 1, a fully connected layer, and a Softmax classification layer.

After several trials, the weights of the network were initialized randomly [20]. The parameters were identified with manual hyperparameter tuning [21,73], using the datasets of two randomly selected subjects. Finally, the network was trained with stochastic gradient descent with momentum, and after several trials, the parameters were set as follows. The learning rate was set to 0.1, L2 regularization to 0.001, momentum to 0.95, batch size to 256, and max epochs of 25.

### 2.7. Statistical Tests

In order to compare the performance of classifiers for all analyses, statistical tests were performed with two-way analysis of variance (ANOVA) using as factors the classifiers and number of days/sessions. A post hoc multiple comparison analysis test [74] was used to compare the performance of individual classifiers. *P* values less than 0.05 were considered significant.

## 3. Results

**Figure 4:**
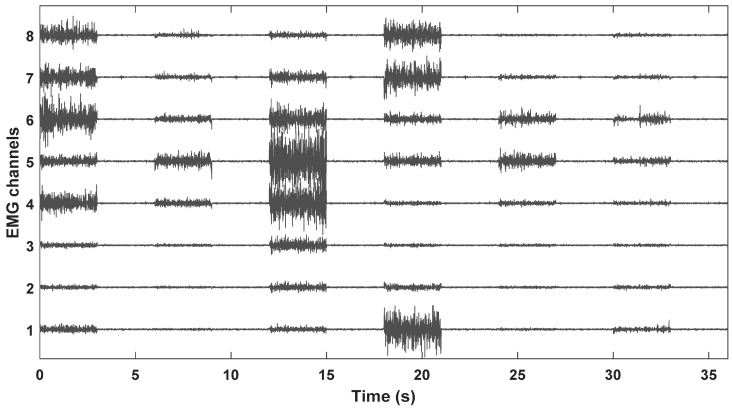
Electromyography (EMG) data recorded via wearable sensors for one repetition from a randomly selected session. The first and last half second of each movement type was removed to avoid transition artifacts. Hence, it shows 3 s of each movement with a rest period of 3 s.

The within-day analyses are presented in Section 3.1 and Section 3.2, while the between-days analyses are presented in Section 3.3 and Section 3.4. The results are presented as mean classification errors (CE) with standard deviation (SD) between subjects. Figure 4 shows the raw EMG data of a randomly selected session for one repetition of each movement along with a rest period, which were directly fed (without any transformation) as inputs to the CNN.

### 3.1. Within-Session Analysis

**Figure 5:**
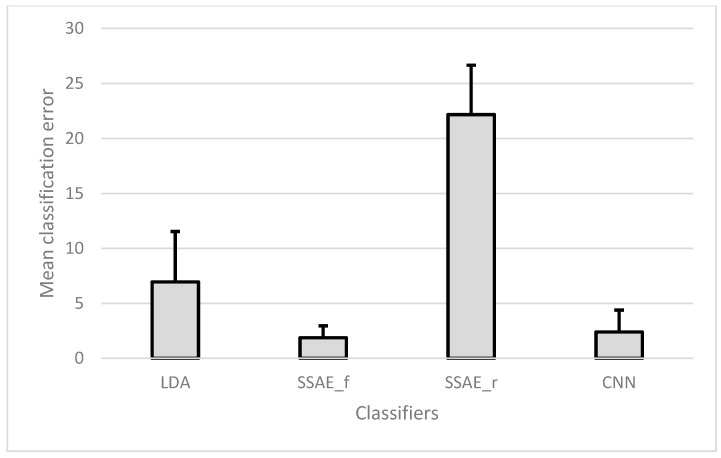
Mean (and SD) classification error of all classifiers for within-session analysis with 10-fold cross-validation averaged over the 15 days. LDA: linear discriminant analysis; SSAE-f: stacked sparse autoencoders with features; and SSAE-r: stacked sparse autoencoders with raw samples.

In this analysis, the CEs of a single session were calculated with 10-fold cross-validation, and they were averaged for 30 sessions of an individual subject. The final results are presented as the mean of all the subjects (Figure 5).

There was no significant difference between SSAE-f and CNN (*p* = 0.55), while both classifiers significantly outperformed (*p* < 0.001) the other two classifiers (LDA and SSAE-r). The large standard deviation (SD) with the classical machine learning technique reveals that there was large difference found in the mean accuracies of the individual subjects. However, this difference was significantly reduced with the proposed techniques (SSAE-f and CNN).

### 3.2. Between-Sessions Analysis

**Figure 6:**
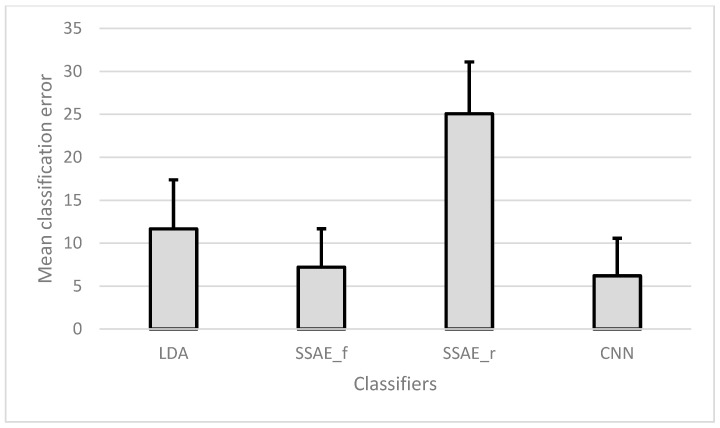
Mean (and SD) classification error of all classifiers for between-sessions analysis with two-fold cross-validation averaged over the 15 days.

In this analysis, two-fold cross-validation was used between sessions completed on the same day. Hence, for the individual subjects, the results of 15 days were averaged, and the final results are presented as the mean CE of all seven subjects, as shown in Figure 6.

No significant difference was found between SSAE-f and CNN (*p* = 0.538), while both the classifiers performed significantly better (*p* < 0.001) than LDA and SSAE_r (worst).

### 3.3. Analysis Between Pairs of Days

For an individual subject, the data of 15 days was organized into 105 unique pairs of days. For each pair of days, two-fold cross-validation was used, and the results of all seven subjects were averaged and are tabulated in Table 1 and Table 2.

In both Table 1 and Table 2, the individual cells represent the mean CE of all the subjects for the corresponding pair of days. Table 1 presents the results with data-driven features, where the upper diagonal is for CNN and the lower for SSAE-r. Table 2 presents the results with handcrafted features, where the upper diagonal is for SSAE-f and the lower for LDA. For an individual subject, the mean CE of this analysis with each classifier is also presented in Table 3.

Overall, LDA, SSAE-f, SSAE-r, and CNN achieved a mean CE ± SD of 14.73 ± 6.32, 10.98 ± 5.47, 25.18 ± 3.93, and 9.79 ± 4.57, respectively. From the statistical tests, it was found that CNN outperformed (*p* < 0.001) all other classifiers, while SSAE-f outperformed (*p* < 0.001) the remaining two classifiers (LDA and SSAE-r).

### 3.4. Leave-One-Out between Days (15-Fold Cross-Validation)

**Figure 7:**
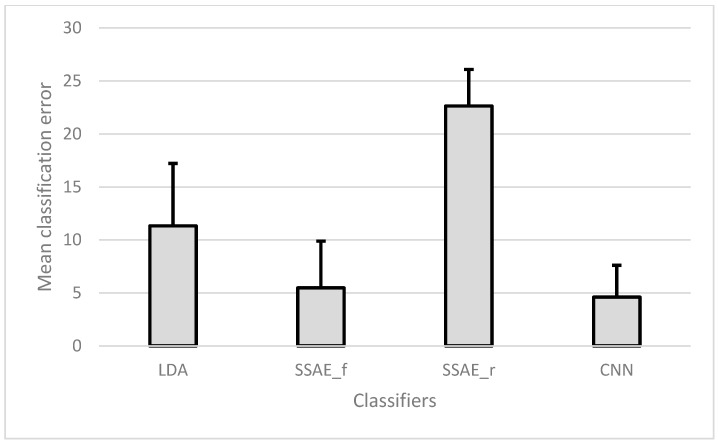
Mean (and SD) classification error of all classifiers for between-days analysis with 15-fold cross-validation.

In this analysis, a 15-fold cross-validation scheme was used, such that each day constitutes a separate fold. The results are presented as the mean CE of all seven subjects, as shown in Figure 7.

Although CNN achieved a comparatively lower error rate than did SSAE-f, there was no significant difference (*p* = 0.219) between the two, and both performed significantly better (*p* < 0.001) than SSAE-r and LDA. In both the between-days analyses, CNN achieved the lowest absolute CE.

### 3.5. Performance of Individual Subjects

Table 3 summarizes the results for each individual subject as the mean CE over 15 days achieved with each classifier in all four analyses.

### 3.6. Computational Time

The classifiers were trained on a system with NVIDIA Quadro k620 GPU (NVIDIA, Santa Clara, CA, USA), a 2.40 GHz processor and 256 GB of RAM. CNN took training and testing times of 13.10, 15.63, 32.43, and 467 s for within-session, between-sessions, pair of days, and between-days analyses, respectively, whereas SSAE-f took 23.44, 26.13, 48.42, and 607.95 s, respectively. SSAE-f achieved higher accuracy than did CNN only in the within-session analysis, while CNN achieved higher accuracies in the rest of the three analyses. Hence, CNN proved to be more robust and computationally efficient than SSAE-f.

## 4. Discussion

We evaluated deep learning techniques (both with EMG features and bipolar samples as inputs) to explore their performances in long-term EMG classification. The main finding is that bipolar EMG is suitable for deep learning when applied to CNN architecture. The performance was not good when applied with SSAE despite the size of the layers. For SSAE, handcrafted features are necessary in similar degrees as classical machine learning, such as LDA.

The results of different analyses reveal that deep learning methods (CNN and SSAE-f) not only outperformed the state-of-art LDA in the short term, but they also showed improved performance over multiple days, and hence, the results were consistent with the notion that deep network performance improves with increasing training size [75]. We hypothesize that with more days (>15), the performance of the leave-one-out analysis will converge towards the within-session performance.

This study explored both handcrafted and data-driven features-based techniques. Based on handcrafted features, classical machine learning algorithms have been widely explored for EMG-based movement classification, and the LDA has emerged as the reference classifier [16,22,76] for commercial systems [66] (https://www.coaptengineering.com). However, in this study, performance measured by CE was poorer with LDA than with autoencoders even in within-session analysis. Moreover, the LDA performance worsened over days. Based on data-driven features, CNN showed promising results as compared with the autoencoders. Although autoencoding generalized well with handcrafted features, it failed to generalize with raw data.

The results of any studies are comparable when there are similar number of classes [21] and the same hardware has been used for recording EMG. Based on the wearable MYB, several studies attempted to classify different wrist movements using either classification errors or accuracy as performance metrics. Mendez et al. [51] classified nine hand movements using the same TDF with LDA and achieved a mean accuracy of 91.67 ± 6.89. Wahid et al. [59] classified three wrist movements using the same TDF with LDA and achieved a mean accuracy of 94.45 ± 5.20 for between-subjects analysis. Masson et al. [61] classified five wrist movements with KNN and achieved an accuracy of 90%. Allard et al. [19] proposed a CNN that used spectrogram of EMG as that input that was recorded in a single session and achieved offline accuracy of 97.8% for seven wrist movements. Our proposed methods (CNN and SSAE-f) achieved mean accuracies of 97.60 ± 1.99 and 98.12 ± 1.07, respectively, in within-session analysis and also showed improved performance over days. The proposed CNN model used bipolar EMG unlike Allard et al. [19], which used a transformed version (spectrogram) of the raw EMG.

The performance of deep learning methods was dependent on the network architecture and optimal parameter selection. For SSAE, the number of units in both layers was optimized so that an increase in units did not significantly improve performance. Similarly, the performance was optimal by using non-linear and linear activation functions for both encoders and decoders respectively. Errors at layers 1 and 2 were dependent on SR and L2R, respectively. For CNN, increased size and number of filters were tested during pilot analyses with no significant improvement, most probably because of the limited sample size and the low complexity of the classification problem. Similarly, the number of epochs was also varied in the preliminary analyses. Some manually tuned parameters, including initial learning rate and momentum, played a significant role. However, varying L2R did not affect the performance. Overall, there was no significant difference between CNN and SSAE-f. However, CNN with bipolar EMG as the input achieved higher accuracies (in three out of four analyses) than did handcrafted features (LDA and SSAE-f). This study presents a preliminary step in the feasibility of reducing the between-days error with increased training size as an effective means of enhancing the long-term robustness of the classification task. However, the study comprised a limited, small number of able-bodied subjects and presented an offline analysis, which limits the possibility of generalizing the results.

## 5. Conclusions

This study demonstrated that deep learning techniques outperformed the classical machine learning algorithm using both handcrafted features and raw EMG signals as inputs. The results of intra/intersessions and between-days analyses imply that CNN has the potential to recognize EMG patterns even from raw bipolar EMG data for long-term classification notwithstanding the stochastic nature of the EMG signals. This is important in mitigating the hassle of feature selection or signal transformation prior to classification. Nevertheless, it should be noted that although CNN performed well with raw EMG, SSAE, which is another deep learning architecture, still requires feature extraction for a robust performance.

## Figures and Tables

**Table 1:** CNN vs SSAE-r. Performance comparison using pairs of days. The upper diagonals show the classification error with CNN for the corresponding pair of days, while the lower diagonal is for SSAE-r. For both CNN and SSAE-r, the classification was performed with bipolar raw EMG data.

Days	01	02	03	04	05	06	07	08	09	10	11	12	13	14	15
**01**	-	5.8	10.25	13.41	13	11.54	12.32	11.82	13.11	11.85	12.5	12.65	12.95	12.87	14.98
**02**	22.71	-	8.16	11.42	9.87	8.97	9.35	8.9	9.76	9.14	9.61	10.48	11.17	10.58	12.13
**03**	26.17	22.89	-	8.97	7.54	8.71	8.24	7.81	9.26	9.91	9.97	11.33	11.7	12	14.33
**04**	29.43	25.05	23.73	-	8.03	11.44	10.59	8.48	8.12	10.88	10.55	12.07	10.47	12.51	12.75
**05**	27.93	23.53	21.95	22.32	-	7.9	7.63	7.33	8.4	7.26	8.1	8.83	10.73	12.54	13.46
**06**	29.55	24.54	24.29	25.48	21.7	-	8.92	8.62	9.12	9.44	10.08	10.47	12.93	12.71	13.65
**07**	29.11	24.52	24.5	25.12	21.88	23.26	-	6.12	8.84	8.79	8.92	10.08	10.51	11.77	13.07
**08**	27.49	22.76	22.94	23.75	20.53	22.34	20.34	-	5.6	7.23	7.73	8.66	8.33	10.5	10.77
**09**	33.03	25.6	27.55	24.31	25.2	25.09	24.58	20.49	-	7.52	9.09	8.29	7.75	9.84	10.11
**10**	30.51	26.29	27.5	28.19	23.82	24.66	24.93	20.68	22.45	-	6.22	6.4	7.33	9.5	9.01
**11**	28.74	24.54	25.82	26.26	22.34	25.06	23.82	20.78	23.25	20.28	-	6.89	7.02	7.77	8.97
**12**	29.53	25.82	27.16	27.18	23.07	24.76	24.11	21.6	22.99	20.5	20.58	-	6.92	8.66	8.6
**13**	30.97	27.91	29.53	27.73	27.01	29.8	27.64	23.27	23.38	21.97	21.06	21.76	-	7.69	8.25
**14**	31.07	27.71	28.59	27.74	26.91	27.94	25.91	22.71	24.14	22.6	21.45	22.24	19.61	-	6.19
**15**	34.4	30.3	32.53	30.32	29.27	30.5	29.94	25.46	25.14	23.41	23.85	23.51	21.36	19.46	-

**Table 2:** SSAE-f vs LDA. Performance comparison using pairs of days. The upper diagonals show the CE with SSAE-f for the corresponding pair of day, while the lower diagonal is for LDA.

Days	01	02	03	04	05	06	07	08	09	10	11	12	13	14	15
**01**	-	6.77	11.54	15.28	13.68	13.08	13.97	13.01	15.55	14.27	14.51	15.31	15.52	18.19	17.95
**02**	9.93	-	8.57	13.02	10.51	10.26	10.07	9.08	10.55	9.88	10.34	11.12	11.29	13.81	14.24
**03**	14.04	11.49	-	9.32	8.37	10.07	8.71	8.18	10.5	10.31	10.7	12.49	12.45	14.73	17.04
**04**	16.97	14.47	13.84	-	9.18	12.29	11.91	9.41	9.96	12.18	11.05	13.2	11.83	15.45	16.36
**05**	16.25	13.85	12.49	12.3	-	7.81	8.24	7.15	9.27	7.54	8.66	9.91	11.52	15.31	16.76
**06**	16.85	13.49	14.54	15.71	12.36	-	8.78	8.77	10.14	10.01	10.54	11.48	14.45	14.05	15.3
**07**	15.57	12.79	13.08	14.54	12.33	12.89	-	6.63	9.09	8.36	8.88	10.13	11.45	13.32	14.29
**08**	15.66	12.85	12.78	13.43	11.9	13.34	11.14	-	6.54	7.25	8.04	8.69	8.55	12.35	12.68
**09**	18.79	14.83	15.68	13.95	13.67	14.68	13.24	10.34	-	7.64	9.41	9.29	9.33	11.47	11.8
**10**	18.66	15.47	15.83	16.72	12.87	14.49	12.99	11.32	12.79	-	6.9	6.9	9.26	11.14	10.68
**11**	17.57	15.03	15.79	15.43	13.4	15.3	12.72	11.82	12.49	11.28	-	7.62	7.84	9.28	10.85
**12**	16.91	15.34	16.91	16.51	13.51	15.54	12.66	11.8	12.38	11.11	10.57	-	7.89	10.22	10.27
**13**	19.07	16.83	17.77	17.48	16.67	18.45	15.55	13.35	13.53	13.91	11.77	11.54	-	9.44	8.85
**14**	20.02	16.95	17.79	17.73	17.09	17.98	15.8	15.1	14.53	16.05	12.41	13.41	12.17	-	7.37
**15**	21.29	18.94	20.85	19.21	19.39	19.73	17.68	15.39	15.32	15.56	13	13.36	12.46	10.16	-

**Table 3:** Mean classification errors of each subject in all four analyses.

Analysis Type	Classifier	Sub 01	Sub 02	Sub 03	Sub 04	Sub 05	Sub 06	Sub 07
**Within-Session**	**LDA**	2.24	5.83	1.73	4.32	10.07	13.5	10.9
	**SSAE-f**	1.08	2.9	1.05	0.7	2.61	1.36	3.4
	**SSAE-r**	18.25	28.69	16.83	19.38	23.42	21.56	27.08
	**CNN**	1.39	4.72	0.68	0.1	2.69	1.83	5.38
**Between-Sessions**	**LDA**	6.28	10.57	4.43	9.4	13.82	16.61	20.54
	**SSAE-f**	3.69	10.56	1.78	4.05	7.02	7.08	16.16
	**SSAE-r**	20.63	30.67	17.06	23.91	24.13	23.95	35.06
	**CNN**	3.39	8.93	1.53	3.52	5.17	6.28	14.52
**Between Pairs of Days**	**LDA**	8.06	14.66	7.63	11.27	16.5	20.33	24.65
	**SSAE-f**	5.43	15.59	6.54	6.8	10.06	12.02	20.39
	**SSAE-r**	20.62	29.21	21.12	24.15	24.39	25.39	31.32
	**CNN**	5.06	14.05	6.24	6.82	8.45	10.23	17.63
**Leave-One-Out Between Days**	**LDA**	5.35	10.3	3.8	8.92	14.58	16.55	19.76
	**SSAE-f**	2.19	6.5	1.56	2.34	5.78	5.7	14.34
	**SSAE-r**	19.95	22.5	24.38	26.15	22.69	19.14	23.64
	**CNN**	2.62	6.96	1.46	2.79	4.48	3.89	10.02

## References

[1] Scott R. Myoelectric control of prostheses: A brief history. Proceedings of the 1992 MyoElectric Controls/Powered Prosthetics Symposium.

[2] Smith L.H., Hargrove L.J. Comparison of surface and intramuscular emg pattern recognition for simultaneous wrist/hand motion classification. Proceedings of the 2013 35th Annual International Conference of the IEEE Engineering in Medicine and Biology Society (EMBC).

[3] Geethanjali P. (2016). Myoelectric control of prosthetic hands: State-of-the-art review. Med. Devices (Auckland NZ).

[4] Hahne J.M., Markovic M., Farina D. (2017). User adaptation in myoelectric man-machine interfaces. Sci. Rep..

[5] Iqbal N.V., Subramaniam K. (2017). A review on upper-limb myoelectric prosthetic control. IETE J. Res..

[6] Scheme E., Englehart K. (2011). Electromyogram pattern recognition for control of powered upper-limb prostheses: State of the art and challenges for clinical use. J. Rehabilit. Res. Dev..

[7] Li G., Schultz A.E., Kuiken T.A. (2010). Quantifying pattern recognition—Based myoelectric control of multifunctional transradial prostheses. IEEE Trans. Neural Syst. Rehabilit. Eng..

[8] Kamavuako E.N., Scheme E.J., Englehart K.B. (2014). Combined surface and intramuscular emg for improved real-time myoelectric control performance. Biomed. Signal Process. Control.

[9] Purushothaman G., Ray K. (2014). Emg based man–machine interaction—A pattern recognition research platform. Robot. Auton. Syst..

[10] Ortiz-Catalan M. (2015). Cardinality as a highly descriptive feature in myoelectric pattern recognition for decoding motor volition. Front. Neurosci..

[11] Hudgins B., Parker P., Scott R.N. (1993). A new strategy for multifunction myoelectric control. IEEE Trans. Biomed. Eng..

[12] Phinyomark A., Phukpattaranont P., Limsakul C. (2012). Feature reduction and selection for EMG signal classification. Expert Syst. Appl..

[13] Phinyomark A., Limsakul C., Phukpattaranont P. (2009). A novel feature extraction for robust EMG pattern recognition. arXiv.

[14] Adewuyi A.A., Hargrove L.J., Kuiken T.A. (2016). Evaluating EMG feature and classifier selection for application to partial-hand prosthesis control. Front. Neurorobot..

[15] Zhai X., Jelfs B., Chan R.H., Tin C. (2017). Self-recalibrating surface EMG pattern recognition for neuroprosthesis control based on convolutional neural network. Front. Neurosci..

[16] Phinyomark A., Quaine F., Charbonnier S., Serviere C., Tarpin-Bernard F., Laurillau Y. (2013). EMG feature evaluation for improving myoelectric pattern recognition robustness. Expert Syst. Appl..

[17] Phinyomark A., Khushaba R.N., Ibáñez-Marcelo E., Patania A., Scheme E., Petri G. (2017). Navigating features: A topologically informed chart of electromyographic features space. J. R. Soc. Interface.

[18] Tkach D., Huang H., Kuiken T.A. (2010). Study of stability of time-domain features for electromyographic pattern recognition. J. Neuroeng. Rehabilit..

[19] Côté-Allard U., Fall C.L., Campeau-Lecours A., Gosselin C., Laviolette F., Gosselin B. Transfer learning for sEMG hand gestures recognition using convolutional neural networks. Proceedings of the International Conference on Systems, Man and Cybernetics.

[20] Xia P., Hu J., Peng Y. (2017). EMG-based estimation of limb movement using deep learning with recurrent convolutional neural networks. Artif. Organs.

[21] Atzori M., Cognolato M., Müller H. (2016). Deep learning with convolutional neural networks applied to electromyography data: A resource for the classification of movements for prosthetic hands. Front. Neurorobot..

[22] Chan A.D., Green G.C. Myoelectric control development toolbox. Proceedings of the 30th Conference Canadian Medical and Biological Engineering Society.

[23] Oskoei M.A., Hu H. (2008). Support vector machine-based classification scheme for myoelectric control applied to upper limb. IEEE Trans. Biomed. Eng..

[24] Sebelius F.C., Rosen B.N., Lundborg G.N. (2005). Refined myoelectric control in below-elbow amputees using artificial neural networks and a data glove. J. Hand Surg..

[25] Chen X., Zhang X., Zhao Z.-Y., Yang J.-H., Lantz V., Wang K.-Q. Hand gesture recognition research based on surface EMG sensors and 2D-accelerometers. Proceedings of the 2007 11th IEEE International Symposium on Wearable Computers.

[26] Wolczowski A., Kurzynski M. (2007). Control of dexterous hand via recognition of EMG signals using combination of decision-tree and sequential classifier. Comput. Recognit. Syst..

[27] Kim J., Mastnik S., André E. Emg-based hand gesture recognition for realtime biosignal interfacing. Proceedings of the 13th International Conference on Intelligent User Interfaces.

[28] Atzori M., Gijsberts A., Castellini C., Caputo B., Hager A.-G.M., Elsig S., Giatsidis G., Bassetto F., Müller H. (2014). Electromyography data for non-invasive naturally-controlled robotic hand prostheses. Sci. Data.

[29] Liu N., Han J., Zhang D., Wen S., Liu T. Predicting eye fixations using convolutional neural networks. Proceedings of the IEEE Conference on Computer Vision and Pattern Recognition.

[30] Collobert R., Weston J. A unified architecture for natural language processing: Deep neural networks with multitask learning. Proceedings of the 25th International Conference on Machine Learning.

[31] Chorowski J.K., Bahdanau D., Serdyuk D., Cho K., Bengio Y. Attention-based models for speech recognition. Proceedings of the 29th Annual Conference on Neural Information Processing Systems 2015.

[32] Min S., Lee B., Yoon S. (2017). Deep learning in bioinformatics. Brief. Bioinform..

[33] Nurse E., Mashford B.S., Yepes A.J., Kiral-Kornek I., Harrer S., Freestone D.R. Decoding EEG and LFP signals using deep learning: Heading TrueNorth. Proceedings of the ACM International Conference on Computing Frontiers.

[34] Acharya U.R., Fujita H., Oh S.L., Hagiwara Y., Tan J.H., Adam M. (2017). Application of deep convolutional neural network for automated detection of myocardial infarction using ECG signals. Inf. Sci..

[35] Narejo S., Pasero E., Kulsoom F. (2016). EEG based eye state classification using deep belief network and stacked autoencoder. Int. J. Electr. Comput. Eng..

[36] Atzori M., Gijsberts A., Heynen S., Hager A.-G.M., Deriaz O., Van Der Smagt P., Castellini C., Caputo B., Müller H. Building the ninapro database: A resource for the biorobotics community. Proceedings of the 2012 4th IEEE RAS & EMBS International Conference on Biomedical Robotics and Biomechatronics (BioRob).

[37] Geng W., Du Y., Jin W., Wei W., Hu Y., Li J. (2016). Gesture recognition by instantaneous surface EMG images. Sci. Rep..

[38] Park K.-H., Lee S.-W. Movement intention decoding based on deep learning for multiuser myoelectric interfaces. Proceedings of the 2016 4th International Winter Conference on Brain-Computer Interface (BCI).

[39] Cun L., Jackel L., Bottou L., Brunot A., Cortes C., Denker J., Drucker H., Guyon I., Muller U., Sackinger E. Comparison of learning algorithms for handwritten digit recognition. Proceedings of the 1st International Conference on Artificial Neural Networks.

[40] Du Y., Jin W., Wei W., Hu Y., Geng W. (2017). Surface EMG-based inter-session gesture recognition enhanced by deep domain adaptation. Sensors.

[41] Du Y., Wong Y., Jin W., Wei W., Hu Y., Kankanhalli M., Geng W. Semi-supervised learning for surface EMG-based gesture recognition. Proceedings of the International Joint Conference on Artificial Intelligence.

[42] Wei W., Wong Y., Du Y., Hu Y., Kankanhalli M., Geng W. (2017). A multi-stream convolutional neural network for sEMG-based gesture recognition in muscle-computer interface. Pattern Recognit. Lett..

[43] Biron K., Englehart K. EMG pattern recognition adaptation. Proceedings of the 18th Congress of the International Society of Electrophysiology and Kinesiology.

[44] Jiang N., Dosen S., Muller K.-R., Farina D. (2012). Myoelectric control of artificial limbs—Is there a need to change focus? [in the spotlight]. IEEE Signal Process. Mag..

[45] He J., Zhang D., Jiang N., Sheng X., Farina D., Zhu X. (2015). User adaptation in long-term, open-loop myoelectric training: Implications for EMG pattern recognition in prosthesis control. J. Neural Eng..

[46] Zia ur Rehman M., Gilani S., Waris A., Niazi I., Slabaugh G., Farina D., Kamavuako E. (2018). Stacked sparse autoencoders for EMG-based classification of hand motions: A comparative multi day analyses between surface and intramuscular EMG. Appl. Sci..

[47] Waris A., ur Rehman M.Z., Kamavuako E.N. Variability of hand motions quantified using EMG root mean square and mean frequency. Proceedings of the International Society of Electrophysiology and Kinesiology.

[48] Huang Q., Yang D., Jiang L., Zhang H., Liu H., Kotani K. (2017). A novel unsupervised adaptive learning method for long-term electromyography (EMG) pattern recognition. Sensors.

[49] Day S. (2002). Important Factors in Surface EMG Measurement.

[50] Lee S., Kruse J. (2008). Biopotential electrode sensors in ECG/EEG/EMG systems. Analog Devices.

[51] Mendez I., Hansen B.W., Grabow C.M., Smedegaard E.J.L., Skogberg N.B., Uth X.J., Bruhn A., Geng B., Kamavuako E.N. Evaluation of the myo armband for the classification of hand motions. Proceedings of the 2017 International Conference on Rehabilitation Robotics (ICORR).

[52] Pizzolato S., Tagliapietra L., Cognolato M., Reggiani M., Müller H., Atzori M. (2017). Comparison of six electromyography acquisition setups on hand movement classification tasks. PLoS ONE.

[53] Phinyomark A., Scheme E. A feature extraction issue for myoelectric control based on wearable EMG sensors. Proceedings of the Sensors Applications Symposium (SAS).

[54] Benatti S., Casamassima F., Milosevic B., Farella E., Schönle P., Fateh S., Burger T., Huang Q., Benini L. (2015). A versatile embedded platform for EMG acquisition and gesture recognition. IEEE Trans. Biomed. Circuits Syst..

[55] Amirabdollahian F., Walters M. Application of support vector machines to detect hand and wrist gestures using a myoelectric armband. Proceedings of the International Conference on Rehabilitation Robotics (ICORR2017).

[56] Arief Z., Sulistijono I.A., Ardiansyah R.A. Comparison of five time series EMG features extractions using myo armband. Proceedings of the 2015 International Electronics Symposium (IES).

[57] Phinyomark A., Khushaba R.N., Scheme E. (2018). Feature extraction and selection for myoelectric control based on wearable EMG sensors. Sensors.

[58] Boyali A., Hashimoto N., Matsumoto O. Hand posture and gesture recognition using myo armband and spectral collaborative representation based classification. Proceedings of the 2015 IEEE 4th Global Conference on Consumer Electronics (GCCE).

[59] Wahid M.F., Tafreshi R., Al-Sowaidi M., Langari R. (2018). Subject-independent hand gesture recognition using normalization and machine learning algorithms. J. Comput. Sci..

[60] Guo W., Sheng X., Liu J., Hua L., Zhang D., Zhu X. Towards zero training for myoelectric control based on a wearable wireless sEMG armband. Proceedings of the 2015 IEEE International Conference on Advanced Intelligent Mechatronics (AIM).

[61] Masson S., Fortuna F., Moura F., Soriano D. São Bernardo do Campo do ABC. Integrating myo armband for the control of myoelectric upper limb prosthesis. Proceedings of the XXV Congresso Brasileiro de Engenharia Biomédica.

[62] Radmand A., Scheme E., Kyberd P., Englehart K. Investigation of optimum pattern recognition methods for robust myoelectric control during dynamic limb movement. Proceedings of the 30th Conference Canadian Medical and Biological Engineering Society.

[63] Montoya M., Henao O., Muñoz J. Muscle fatigue detection through wearable sensors: A comparative study using the myo armband. Proceedings of the XVIII International Conference on Human Computer Interaction.

[64] Zhang Y., Chen Y., Yu H., Yang X., Lu W., Liu H. (2018). Wearing-independent hand gesture recognition method based on EMG armband. Pers. Ubiquitous Comput..

[65] Côté-Allard U., Fall C.L., Drouin A., Campeau-Lecours A., Gosselin C., Glette K., Laviolette F., Gosselin B. (2018). Deep learning for electromyographic hand gesture signal classification by leveraging transfer learning. arXiv.

[66] Bellingegni A.D., Gruppioni E., Colazzo G., Davalli A., Sacchetti R., Guglielmelli E., Zollo L. (2017). NLR, MLP, SVM, and LDA: A comparative analysis on EMG data from people with trans-radial amputation. J. Neuroeng. Rehabilit..

[67] Kamavuako E.N., Scheme E.J., Englehart K.B. (2016). Determination of optimum threshold values for EMG time domain features; a multi-dataset investigation. J. Neural Eng..

[68] Le Q.V. (2015). A tutorial on deep learning part 2: Autoencoders, convolutional neural networks and recurrent neural networks. Google Brain.

[69] Ur Rehman M.Z., Gilani S.O., Waris A., Niazi I.K., Kamavuako E.N. A novel approach for classification of hand movements using surface EMG signals. Proceedings of the 2017 IEEE International Symposium on Signal Processing and Information Technology (ISSPIT).

[70] Ur rehman M.Z., Gilani S.O., Waris A., Jochumsen M., Niazi I.K., Kamavuako E.N. Performance of combined surface and intramuscular EMG for classification of hand movements. Proceedings of the 2018 40th Annual International Conference of the IEEE Engineering in Medicine and Biology Society (EMBC).

[71] Møller M.F. (1993). A scaled conjugate gradient algorithm for fast supervised learning. Neural Netw..

[72] Bengio Y., Lamblin P., Popovici D., Larochelle H. Greedy layer-wise training of deep networks. Proceedings of the 21th Annual Conference on Neural Information Processing Systems.

[73] Goodfellow I., Bengio Y., Courville A., Bengio Y. (2016). Deep Learning.

[74] McHugh M.L. (2011). Multiple comparison analysis testing in ANOVA. Biochem. Med. Biochem. Med..

[75] Dean J., Corrado G., Monga R., Chen K., Devin M., Mao M., Senior A., Tucker P., Yang K., Le Q.V. Large scale distributed deep networks. Proceedings of the 26th Annual Conference on Neural Information Processing Systems.

[76] Linderman M., Lebedev M.A., Erlichman J.S. (2009). Recognition of handwriting from electromyography. PLoS ONE.

